# Serpins show structural basis for oligomer toxicity and amyloid ubiquity

**DOI:** 10.1016/j.febslet.2008.06.021

**Published:** 2008-07-23

**Authors:** Robin W. Carrell, Alec Mushunje, Aiwu Zhou

**Affiliations:** a Department of Haematology, Cambridge Institute for Medical Research, University of Cambridge, Cambridge CB2 2XY, UK; b Department of Medicine, Cambridge Institute for Medical Research, University of Cambridge, Cambridge CB2 2XY, UK

**Keywords:** Oligomers, Amyloid, Serpin, Alzheimers, Prions

## Abstract

Many disorders, including Alzheimer’s, the prion encephalopathies and other neurodegenerative diseases, result from aberrant protein aggregation. Surprisingly, cellular toxicity is often due not to the highly-ordered aggregates but to the oligomers that precede their formation. Using serpins as a paradigm, we show how the active and infective interface of oligomers is inherently toxic and can promiscuously bind to unrelated peptides, including neurotransmitters. Extension of the oligomer and its eventual sequestration as amyloid can thus be seen as a protective response to block the toxic interface. We illustrate how the preferential self-association that gives this protection has been selectively favoured.

## 1 Introduction

The protein aggregation that underlies conformational diseases such as the prevalent Alzheimer’s, Parkinson’s and prion encephalopathies [1–4], is due not to random addition but to sequential β-interlinkages resulting in progressive fibral elongation [5,6]. The end-point of these progressive β-linkages is the formation of highly-ordered fibrillar and amyloid deposits. The presence in many of the conformational diseases of readily recognisable amyloid deposits led to the presumption that the diseases themselves were due to the formation of amyloid. The massive deposition of amyloid can indeed result in organ failure but for the most part and particularly with the neurodegenerative diseases, the appearance of amyloid deposits is a late and inconsistent feature. Similarly the accumulation of long-chain protein polymers can directly affect cellular viability [7] but there is now increasing evidence that cellular damage and specifically neurotoxicity often arises much earlier in the disease process, at the stage of initial oligomer formation [8–12]. The generality of this unexpected finding [13]. indicates a shared mechanism. This has focused our attention on the one mechanistic feature common to all the conformational diseases, the formation of the intermolecular β-bonding responsible for the protein aggregation.

**Fig. 1:**
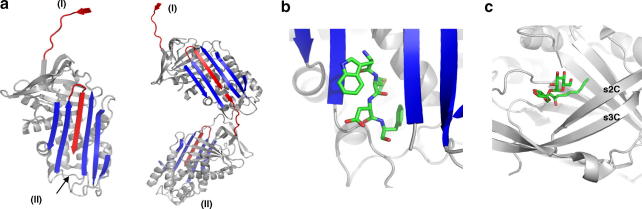
Serpin β-interfaces and linkages. Crystallographic depictions: (a) Cleavage of the reactive centre loop (red) of α1-antitrypsin creates a β-donor strand I, and an accompanying opening of the A-sheet (blue) creates a β-acceptor site II. Subsequent interlinkage, as shown with the dimer, re-forms each of these sites as the oligomer extends (PDB 1QMB) [15]. (b) The hexapeptide acceptor site II seen here in a polymerogenic form of antithrombin [25], can promiscuously bind other peptides including as shown the neurotransmitter peptide CCK4 (PDB 1JVQ). But even the peptide that naturally occupies this site, TAVVIA, has to be in a 100-fold excess to compete with the preferential linkage to the site of another molecule of antithrombin. (c) The potential promiscuity and hence toxicity of a vacated β-acceptor site is seen here with exposure of the s1C strand position in α1-antitrypsin (see also Fig. 3a) and the binding to it of a liposaccharide from Im et al (PDB 1IZ2) [30].

To determine why early oligomers formed by such linkages are consistently toxic we have examined the β-interlinkages formed by members of a widely distributed family of serine protease inhibitors, the serpins [6,14]. The advantage of using the serpins as a model is that the mechanism of their fibrillar aggregation is known in crystallographic detail [15,16]. The inhibitory efficiency of the serpins is dependent on the ability of their cleaved reactive–centre peptide loop to insert into the middle-strand position of the 5-stranded β-sheet A of the molecule. This ability of the A β-sheet to undergo a transition from a 5- to a 6-stranded form makes the serpins susceptible to intermolecular linkages, as an aberrant opening of the A-sheet allows the insertion into it of the reactive loop of another molecule (Fig. 1). The sequential formation of similar domain exchanges in a number of other proteins likewise results in oligomer and polymer formation in a process descriptively summarised as ‘runaway domain swapping’ [17]. This notably occurs with the plasma serpin α1-antitrypsin that protects the lungs against the proteases released by inflammatory cells. People of European descent commonly carry the unstable Z-variant of α1-antitrypsin that readily forms loop-sheet polymers. The polymerisation of Z-antitrypsin [18] principally takes place at its site of synthesis in the hepatocyte and its accumulation there, as intracellular inclusions, is in itself damaging and leads to the eventual development of liver cirrhosis. By a precisely similar process, mutations in a neurone-specific serpin can result in its intracellular polymerisation in neurones leading to an Alzheimer-like late-onset encephalopathy and dementia [7,19].

**Fig. 2:**
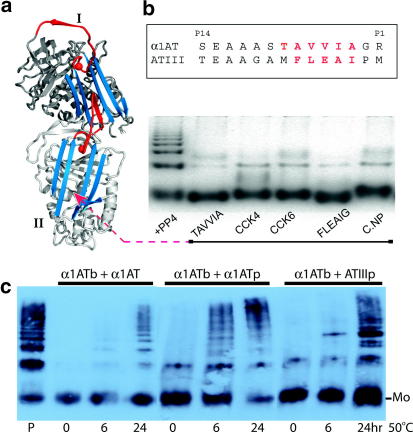
Oligomer extension and toxicity. (a) Oligomer formation in the *intact* serpins results from the formation of an initiating dimer [20] shown diagrammatically here, labelled as in the *cleaved* dimer in Fig. 1a. (b) Native PAGE (and table of reactive centre loop sequences). Polymerisation of plasma α1-antitrypsin (α1AT) is readily induced [15,22] by loop-cleavage with the protease PP4 but is blocked by incubation [25] at 37 °C of a 100-fold molar excess of the peptide FLEIAG that normally occupies this strand vacancy in α1-antitrypsin, but also equally effectively not only by the homologous peptide TAVVIA from antithrombin (ATIII) but also by the cholecystokinin peptides CCK4 and CCK6 and the cardioexcitatory neuropeptide C.NP. (c) Native PAGE of biotin-labelled α1-antitrypsin (α1ATb) at 0.1 mg/ml incubated at 50 °C for 0–24 h and visualised with a chemiluminescent substrate [20]. P, polymer marker and left three lanes with control unlabelled α1-antitrypsin (0.5 mg/ml). Middle three lanes show that addition of 0.5 mg/ml of pre-formed α1-antitrypsin polymers (α1ATp) results in the polymerisation and consumption of the labelled monomer. A similar addition of 0.5 mg/ml polymers (ATIIIp) of antithrombin in the three lanes on right shows that heterogeneous interlinkage does occur but not to an extent that discernibly consumes the monomeric α1-antitrypsin (Mo).

We have recently shown [20] that the initiating step in serpin polymerisation occurs when two molecules with coincidentally perturbed conformations link to form an initial dimer with two active interfaces (Figs. 1 and 2a). In one unit of the dimer the A-sheet is stabilised in a partially opened form, making it an activated acceptor for β-strand linkages, whereas the other unit has an exposed reactive centre peptide loop held in a constrained conformation optimal for β-strand donation. Once it is formed this nucleating and infective oligomer can then recruit further molecules resulting in a sequential re-formation, molecule by molecule, of each of the active interfaces [20]. We show here, with the serpins α1-antitrypsin and antithrombin, how the exposed β-acceptor interface of such oligomers is potentially toxic in that it can promiscuously link to neurotransmitter and other vital peptides. This toxicity will be limited however by the competitive blocking of the site by auto-linkages resulting in the progressive extension of the oligomer and its eventual sequestration. An indication as to why self-association is so competitively successful comes from the demonstration here of a β-bonding interface in antithrombin that is constantly being exposed in blood but is immediately and preferentially blocked by a specific auto-linkage.

## 2 Materials and methods

### 2.1 Materials

Human α-antithrombin (ATIII) and α1-antitrypsin (α1AT) were purified from frozen plasma as previously described [18,21]. Polymers of ATIII or α1AT were prepared by heating the protein at 60 °C for 15 min at 1 mg/ml at pH7.4 or by PP4 protease cleavage [22]. Human α1AT mutants with P7-P3 of the reactive loop substituted to AVVIA (P7-P3 of the reactive loop of ATIII) or VTFKA (strand 1 of C-sheet of ATIII) were prepared from *E. coli* and purified using nickel-chelating and ionic exchange HiTrap Q columns (GE Healthcare) as previously described [23]. Peptides CCK4 (WMDF), CCK6 (DYMGWM) and C.NP, the cardioexcitatory neuropeptide (FLRF),were from Sigma–Aldrich Ltd., Dorset, England. Latent antithrombin and its dimeric derivatives were prepared and characterised as previously described [21,24].

### 2.2 Biotin labelling

To label the only cysteine residue of α1AT with biotin, plasma α1AT was first treated with 10 mM DTT, pH 7.4, at room temperature for 15 min and purified from free DTT by a NAP10 desalting column. A freshly-made solution of *N*-(3-maleimidylpropiony)biocytin (Molecular Probes) was added to the protein solution (∼1 mg/ml) at 10 molar excess and the mixture was kept at room temperature for 2 h. The reaction was stopped by adding 10 mM DTT and the labelled α1AT was purified by a Hitrap Q column. To detect the biotin labelled protein, samples were analysed by native gel electrophoresis, and then transferred to a nitrocellulose membrane, blotted with streptavidin-peroxidase polymer (Sigma) and visualised using the ECL Western Blotting Detection Regents (GE healthcare). Native gel electrophoresis was performed as previously described using an 8% polyacrylamide gel [24].

## 3 Results and discussion

### 3.1 Toxic interface

The potential toxicity of the activated β-interface of early oligomers, is demonstrated in Fig. 2b with the polymerisation of α1-antitrypsin induced by cleavage of its reactive loop by the protease PP4 [15,16,22]. This shows the relatively non-specific amino acid sequence requirement for β-strand linkage to the acceptor interface, with polymerisation being blocked not only by the annealing of the β-strand peptide that normally occupies the acceptor interface in α1-antitrypsin (FLEAI) but also, with even greater efficiency, by the equivalent but quite different peptide from antithrombin (TAVVIA) [25]. As shown the interface can also bind with equal efficiency to a range of other small peptides, including the cholecystokinin neuropeptides *CCK6* (DYMGWM) and *CCK4* (WMDF) and the cardioexcitatory neuropeptide *C.NP* (FLRF). The ability to bind to heterogeneous peptide sequences in this way is not confined to the polymers of α1-antitrypsin induced by loop cleavage but also occurs with the heat-induced oligomers of polymerogenic forms of antithrombin and with mutant Z α1-antitrypsin [25]. This inherent ability of oligomers to promiscuously form β-linkages with a range of peptides, explains their potential toxicity. Although this potential toxicity is demonstrated here with the ready ability to bind neurotransmitter and other peptide messengers, lethal damage is more likely to occur within the milieu of the cell due to a similar binding to the peptide loops of receptors [26] or of other key cellular and membrane components [27]. But protection against such promiscuous and potentially lethal linkages will be provided by the much more competitively avid formation of auto-linkages.

### 3.2 Specificity of auto-linkages

Although we show in Fig. 2b the blockage of polymer extension by various peptides, unless the competing peptide is present in greater than 50:1 molar ratio with respect to the monomer[25,28] serpin oligomers will preferentially link with further serpin monomers to give oligomeric extension and polymerisation. Moreover the auto-linkage is relatively specific for each serpin. As shown in Fig. 2c, monomers of α1-antitrypsin readily link to pre-formed polymers of α1-antitrypsin but only detectably so to polymers of the closely related serpin, antithrombin. Such preferential auto-linkages are readily explicable if they involve major domain exchanges but the surprising finding is that preferential auto-linkage also takes place even when the linkage involves only a small 6-residue peptide sequence, as with the polymerogenic serpins depicted in Fig. 1a and b. We conclude that the interlinkage of serpins is dependent not only on strand sequences but also requires a conformational flexibility - as induced by thermal (50 °C) stress or as occurs spontaneously, at 37 °C, with the unstable variants associated with disease. Hence the aggregates of α1-antitrypsin in the hepatocytes that cause the associated cirrhosis are homogeneous and contain only the abnormal Z-variant and similarly the neuronal aggregates in the neuroserpin encephalopathies contain only the variant neuroserpin despite the presence of the normal allelic protein.

**Fig. 3:**
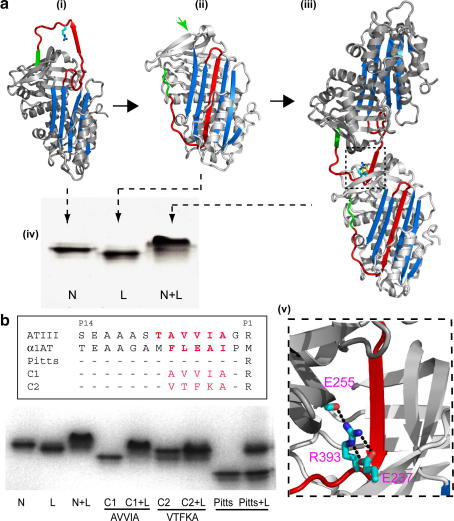
Specificity of autolinkage and blockage of a β-interface. (a) The senescent transition of active antithrombin (i) to its latent form (ii) exposes a vacated strand position (green, arrowed), see also Fig. 1c. This is immediately filled (iii) by the reactive loop, in red, of an active antithrombin molecule to give formation of a heterodimer as seen (iv) on native-PAGE showing the addition of active antithrombin (N) to equimolar latent antithrombin (L) [21,24]. (b) Native-PAGE showing dimer formation with latent antithrombin and recombinant variants of α1-antitrypsin (Table). The Pittsburgh variant of α1-antitrypsin with a reactive centre arginine (Pitts) does not form a dimer if added to equimolar latent antithrombin, nor does it if the sequence VTFKA of the displaced strand is inserted in the reactive centre loop (C2). But dimer formation does occur (C1) when this sequence is replaced by that present in antithrombin AVVIA. The structural basis for this specificity of interlinkage and hence of the blocking of the exposed β-acceptor site in latent antithrombin is indicated in the inset (v) expanded from (iii). This shows [31] the bridging of the two molecules by the reactive centre arginine 393 as well as by the AVVIA strand (red) of the reactive centre loop of the active molecule.

A clear example of how the specificity of this self-association has been influenced by selective design comes from observations of another conformational change in a plasma serpin (Fig. 3). Each day, in everyone, some 4% of the total circulating antithrombin undergoes a senescent conformational transition to an inactive ‘latent’ form with a 6-stranded A-sheet [21,24,29]. This addition of an extra strand to the A-sheet is accompanied by the removal of the outer strand from another β-sheet of the molecule, the C-sheet. The potential for this vacant and exposed β-strand position in the C-sheet to form potentially toxic linkages is seen with α1-antitrypsin in Fig. 1c [30]. In antithrombin however, the vacant β-strand position is immediately filled and blocked by linkage to it of the reactive centre loop of an active molecule of antithrombin to give an active-latent heterodimer (Fig. 3a, iii). The sequence of the peptide section (AVVIA) of the reactive loop of active antithrombin that occupies the vacated site in the C-sheet in the latent molecule bears no apparent relationship to that of the displaced strand (VTFKA). This then seems a relatively non-specific linkage and we fully expected that the addition of purified latent antithrombin to plasma would result in a number of similarly non-specific linkages to other plasma proteins. But to our surprise the only detectable linkage, which accounted for all the added latent antithrombin, was to active plasma antithrombin. To further examine the specificity of this linkage we incubated latent antithrombin with a series of other plasma serpins all of which have exposed peptide loops. A weakly linked dimer was formed with antichymotrypsin that was readily displaced by antithrombin (not shown) but there was no linkage with other serpins including α1-antitrypsin. The specificity of linkage was also tested using the ‘Pittsburgh’ variant of α1-antitrypsin in which the reactive centre methionine of α1-antitrypsin is replaced by an arginine as is present in antithrombin (Fig. 3b). The wild-type antitrypsin-Pittsburgh did not form a dimer with latent antithrombin but dimers were formed when the reactive centre loop of the Pittsburgh antitrypsin was homologously modified to the AVVIA sequence present in the reactive loop of antithrombin but not when the sequence was modified to the VTFKA of the displaced C-sheet strand. The explanation for these findings is clear on examination of the crystal structure of the heterodimer [31]. The intermolecular linkage between the latent and active molecules involves a number of contacts outside and beyond those formed by the replacement strand. In particular the reactive centre arginine present in antithrombin, and also in antitrypsin-Pittsburgh, forms a pair of hydrogen bonds that link the two molecules with a structural specificity that is persuasively purposeful (Fig. 3a, v). Thus as soon as the latent molecule is formed a specific interaction with an active molecule of antithrombin effectively blocks the exposed β-interface, pending the catabolic removal of the senescent molecule. Although this may be an extreme example, the homogeneity of the oligomers formed by other serpins (Fig. 2c) similarly indicates a preferential and inherent conformational complementarity.

### 3.3 Why is amyloid so ubiquitous?

The presence of amyloid deposits is such a frequent and evident finding in the conformational diseases that they have often been referred to collectively as the amyloidoses. But if amyloid is not the toxic species, why then is its presence so ubiquitous? An accompanying puzzle for protein chemists is why, if β-linkages are non-demanding, is amyloid so homogeneous? It is no surprise that purified proteins, if unfolded in vitro, form ordered and sequential intermolecular β-linkages. The puzzle comes from the almost universal finding that even in the complex mixtures that exist in vivo there is still a specific and homogeneous self-association. This will in part reflect the requirement for domain complementarity but as we illustrate here, there has also been a selective adaptation of the structures of conformationally vulnerable molecules to favour intermolecular linkage, as a defensive response to the exposure of active and potentially toxic β-interfaces. Not only does antithrombin selectively and specifically dimerise to protect a vacated interface (Figs. 1c and 3a) but in the serpins in general, exposure of a hexapeptide β-acceptor site (Fig. 1b) is competitively filled by auto-linkages in a 50:1 preference to the natural hexapeptide (Fig. 2b). This ability to preferentially form auto-linkages enables the early oligomer to scavenge and selectively remove molecules that may, even transitorily, change their fold. The Conformational Diseases result from a great diversity of oligomeric linkages - a diversity seen even within the serpins which have at least five different identified inter-strand linkages [14] only two of which, to strands 1C and 4A, have been illustrated here. The wider relevance of the findings from the serpins is the demonstration, in structural detail, of the consequences of the formation of active *β*-acceptor and donor interfaces. Although we demonstrate this with the domain exchange and small-peptide interlinkage of ordered proteins the same will apply with the oligomers formed by individual free peptides, where recruitment similarly occurs, molecule by molecule, to an extendable terminal β-interface [32].

These simple concepts explain why fibrillar deposits and amyloid are such a ubiquitous feature of the conformational diseases. The deposits can now be seen as the end-point of a protective response [13,33] that sequestrates active and potentially toxic interfaces. The ability of the oligomers to recruit and extend also explains their inherent infectivity, with the fragmentation of polymers initiating further rapid polymerisation [34]. The conformational changes that are induced in the serpins on oligomer formation are reversible so the infectivity is self-limiting. But a special danger will arise if the induced conformational changes are irreversible, as with prions, to give a progressive and cumulative infectivity. The overall conclusions also have implications for approaches to treatment. A priority target for therapy is the blockage of the activated and hence potentially toxic interface of the early oligomer. Paradoxically, there may be circumstances where it is instead advantageous to encourage oligomer extension in order to sequestrate the β-interface.
